# Heart rate in patients with reduced ejection fraction: relationship between single time point measurement and mean heart rate on prolonged implantable cardioverter defibrillator monitoring

**DOI:** 10.1186/s12872-018-0751-2

**Published:** 2018-01-31

**Authors:** Marlena V. Habal, Kumaraswamy Nanthakumar, Peter C. Austin, Cassandra Freitas, Christopher Labos, Douglas S. Lee

**Affiliations:** 1 University of Toronto, Toronto, ON Canada; 2 Peter Munk Cardiac Centre, University Health Network, Toronto, ON Canada; 3 Institute for Clinical Evaluative Sciences, Toronto, ON Canada; 4 Ted Rogers Centre for Heart Research, Toronto, ON Canada; 5 University Health Network, Toronto General Hospital, 200 Elizabeth Street, 4NU-482, Toronto, ON M5G 2C4 Canada

**Keywords:** Mean heart rate, Resting heart rate, Beta-blockers

## Abstract

**Background:**

Heart rate (HR) is a prognostic marker that is increasingly used as a therapeutic target in patients with cardiovascular disease. The association between resting and mean HR remains unclear. We therefore set out to determine the relationship between resting HR on the electrocardiogram (ECG) obtained at a single time point, and mean HR on implantable cardioverter defibrillator (ICD) interrogation amongst patients with a reduced left ventricular ejection fraction (LVEF).

**Methods:**

Prospective ICD data were obtained from 54 patients with LVEF < 40%. Mean HR determined using the ICD HR histograms was compared with resting HR measured on the ECG performed in the clinic.

**Results:**

Average resting and ICD mean HRs were 67.9 ± 10.1 and 67.8 ± 9.6 bpm respectively. There was good correlation in the overall cohort (*r* = 0.79), in those with resting ECG HRs ≤ 70 bpm (*r* = 0.62), and amongst the 27 patients on intermediate-to-high dose beta-blockers (*r* = 0.91). However, Bland-Altman analysis demonstrated wide limits of agreement in the overall cohort (− 12.5, 12.7 bpm), at resting HRs ≤ 70 bpm (− 12.7, 9.8 bpm), and on intermediate-to-high dose beta-blockers (− 8.9, 7.4 bpm). Moreover, resting HR did not predict the 10-bpm interval where the most time was spent.

**Conclusions:**

While resting HR correlated with mean HR in patients with reduced LVEF, and in important subgroups, the limits of agreement were unacceptably wide raising concern over the use of single time point resting HR as a therapeutic target.

**Electronic supplementary material:**

The online version of this article (10.1186/s12872-018-0751-2) contains supplementary material, which is available to authorized users.

## Background

Elevated heart rate (HR) has been associated with higher mortality in otherwise healthy individuals [1–3] and in those with coronary artery disease [4, 5]. In the Framingham cohort, higher HR was associated with an increased risk of cardiovascular disease, heart failure, and death [6]. Our group and others have previously shown that an elevated HR is associated with increased all-cause and cardiovascular mortality as well as with 30-day re-hospitalization in chronic heart failure [7–9]. The negative effect of elevated HR on the myocardial supply-demand balance is implicated in this process and clinically, elevated HR has been associated with progression of atherosclerosis in patients with an MI at a young age [10] and plaque disruption [11].

Ivabradine, an I_f_ current inhibitor that selectively lowers HR was initially shown to improve time to ST-segment depression in patients with stable angina [12]. More recently, ivabradine was studied in the heart failure (HF) population where it reduced HF hospitalizations (SHIFT) [13] resulting in its inclusion as part of guideline recommended therapy for HF patients with HR ≥ 70 bpm despite optimal medical therapy [14, 15]. However, despite the growing interest in heart rate modulation as a target for therapeutic intervention, there is a paucity of literature on the optimal method of HR assessment.

While resting HR is commonly used in the clinical setting due to its simplicity, it has been shown that single time-point HR measurement reproducibility is poor with only modest correlation between resting HR and mean HR obtained on extended monitoring [16–18]. Moreover a recent study examining ambulatory monitoring suggested that continuous HR was an independent predictor of all-cause mortality after adjusting for resting HR while adjusted resting HR was not [19]. This has implications not only from a therapeutic perspective but also merits investigation to inform clinical trial design. Indeed, the ivabradine trials have shown mixed results both in the setting of ischemic heart disease [12, 20], and chronic HF [13, 21]. Therefore we sought to examine the relationship between resting and mean HR on prolonged monitoring in patients with reduced LVEF. However, because correlation does not necessarily imply agreement, we further sought to investigate the level of agreement between these measures in order to ascertain the safety and validity of these methods of HR determination.

## Methods

### Study population and study design

We prospectively recruited a convenience sample of patients with systolic dysfunction between April 2015 and March 2016 who were followed in the device clinic at Toronto General Hospital, a large tertiary care centre in the University Health Network, Toronto, Canada. Patients were eligible for inclusion if they were older than 18 years old, had an LVEF less than 40%, had an ICD in situ, and were paced in the atrium or ventricle less than 20% of the time. Patients with an LVEF greater than or equal to 40%, who were paced more than 20% of the time, who had a HR less than 40 bpm, or a paced rhythm on resting rhythm strip during their clinic visit were excluded. Patients with a left ventricular assist device or complex congenital heart disease were also excluded.

Baseline characteristics and device indication were obtained from the electronic patient record (EPR). Medication lists were obtained directly from the patient, or if not available, from the chart or EPR. Analyses were limited to those patients whose medication lists were documented within 13 weeks of HR assessment. Beta-adrenoreceptor blockers were stratified into low and intermediate-high dose categories. Carvedilol ≥12.5 mg twice daily, bisoprolol ≥5 mg daily, and metoprolol ≥ 50 mg twice daily were considered to be intermediate-high doses. The intermediate-high dose category which we analyzed together, is consistent with high dose group in the recent OBTAIN study [22].

### Ascertainment of heart rate

Resting HR was defined as that obtained from the device interrogation at the end of the clinic visit in the seated or recumbent position after the patient had been resting both prior to and during the clinic visit. Mean HR was obtained from the HR histogram available upon device interrogation. The HR histogram is divided into 10 bpm heart rate intervals, (for example 60–69 bpm, 70–79 bpm, etc.) and provides the relative amount of time spent in each heart rate interval as a percentage of the total time since last device interrogation. Mean HR was calculated by taking the median of each 10-bpm heart rate interval and multiplying it by the percentage of time spent in that interval. The mean HR was the sum of the products calculated in each interval. The highest % HR interval was defined as the 10-bpm interval that the patient spent the most time in. If multiple 10-bpm intervals had equal frequencies, the one closest to the resting HR was used. An illustrative example of our calculation of mean heart rate can be seen in Additional file 1: Figure S1.

### Outcomes

The primary goal was to determine the agreement between resting HR on the ECG at a single time point and mean HR from the ICD interrogation. Secondary analyses included the correlation between resting HR and the highest % HR interval. We also conducted a sensitivity analysis to examine the relationship in important subgroups including HR ≤ 70 bpm, diabetics, patients with arrhythmias on interrogation (atrial fibrillation and ventricular tachycardia), those taking intermediate-high doses of beta-blockers, the subgroups with and without pacing on interrogation, and those with cardiac resychronization therapy (CRT).

### Statistical analysis

Continuous variables are reported as mean ± standard deviation and categorical variables as numbers (percentages). The correlation coefficient (r) was determined for the association between resting HR and mean HR, as well as the association between resting HR and the highest % HR interval. Percent agreement between the two HR measurements was assessed using Bland-Altman plots with 95% limits of agreement. Statistical analysis was performed using STATA version 12, StataCorp, College Station, Texas.

## Results

### Patient characteristics

A total of 54 patients were included in the present study of which 41 (76%) were male. The average age was 63.6 ± 12.6 years. The baseline characteristics are shown in Table 1. Twenty-four patients (44%) had an LVEF between 20 and 29% and 32 (59%) had an ischemic etiology for their cardiomyopathy. With regards to the ICD indication, 41 devices (76%) were for primary prevention. Nineteen patients (35%) were paced > 1% in the chamber used to quantify heart rate (ie. excluding CRT) with an overall mean percentage time paced of 6.6%.

**Table 1:** Baseline Characteristics

Age (years)	63.6 ± 12.6
Male, n (%)	41 (76)
Indication for ICD, n (%)	
1^o^	41 (76)
2^o^	13 (24)
Ischemic HF, n (%)	32 (59)
LVEF, n (%)	
30–39	16 (30)
20–29	24 (44)
< 20%	14 (26)
Comorbidities, n (%)	
Hypertension	29 (54)
Dyslipidemia	30 (56)
Diabetes	20 (37)
Device, n (%)	
Medtronic	15 (28)
St. Jude	34 (63)
Boston	4 (7)
Biotronik	1 (2)
Dual chamber, n (%)	36 (67)
CRT, n (%)	22 (41)
Paced> 1%, n (%)	19 (35)
Mean time paced	6.6%
Atrial fibrillation history, n (%)	16 (30)
Atrial fibrillation on interrogation, n (%)	15 (28)
History of VT/VF, n (%)	30 (56)
VT on interrogation, n (%)	6 (11)
NSVT on interrogation, n (%)	13 (24)
Medications, n(%)^a^	
Beta-blockers	39 (100%)
Amiodarone	7 (18%)
Digoxin	12 (31%)
ACEI or ARB	37 (95%)
Mineralocorticoid antagonist	29 (74%)
Furosemide	24 (62%)

*ICD* Implantable Cardioverter Defibrillator, *HF* heart failure, *LVEF* left ventricular systolic function, *CRT* cardiac resynchronization therapy, *VT* ventricular tachycardia, *VF* ventricular fibrillation, *NSVT* nonsustained ventricular tachycardia

^a^medication data were known and available for 39 patients

### Association between resting and mean heart rate

**Fig. 1:**
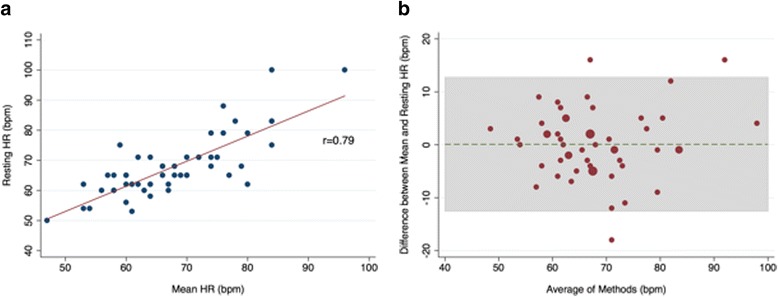
**a** Resting HR/mean HR. **b** Bland-Altman Plot of resting HR/mean HR. HR, heart rate

For the total study population, the mean resting HR was 67.9 ± 10.1 bpm and the ICD derived mean HR was 67.8 ± 9.6 bpm. There was good linear correlation (*r* = 0.79; *p* < .001) between these two measurements (Fig. 1a). Using Bland-Altman analysis, the mean difference was minimal (0.1). However the limits of agreement were wide (− 12.5, 12.7 bpm) (Fig. 1b). When the limits of agreement were set to ±5 bpm, 20 (37%) of the values fell outside of this range (Additional file 2: Figure S2).

### Association between resting heart rate ≤ 70 bpm and mean heart rate

**Fig. 2:**
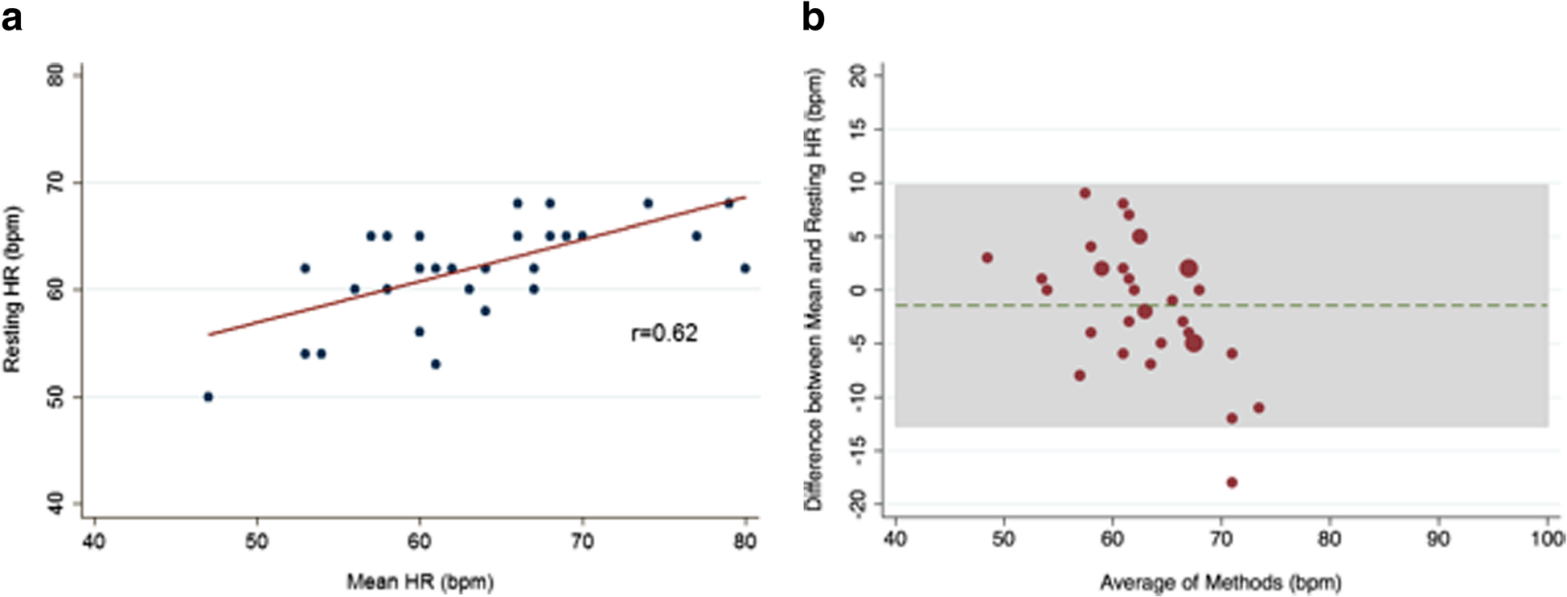
**a** Resting HR/mean HR amongst the subgroup with resting HR ≤ 70 bpm. **b** Bland-Altman Plot of resting HR/mean HR amongst the subgroup with resting HR ≤ 70 bpm

The association between resting and mean HR in the subgroup with resting HR ≤ 70-bpm is presented in Fig. 2a. Moderate correlation (*r* = 0.62; *p* < .001) was found in this subgroup of patients. Bland-Altman analysis demonstrated a slight difference between resting and mean HR (−1.4 bpm) (Fig. 2b). Similar to the overall cohort, the limits of agreement were wide (−12.7, 9.8 bpm). When the limits of agreement were set to ±5 bpm, 11 (30.6%) of the values fell outside of this range.

### Association between resting heart rate and mean heart rate in patients prescribed beta-blockers

**Fig. 3:**
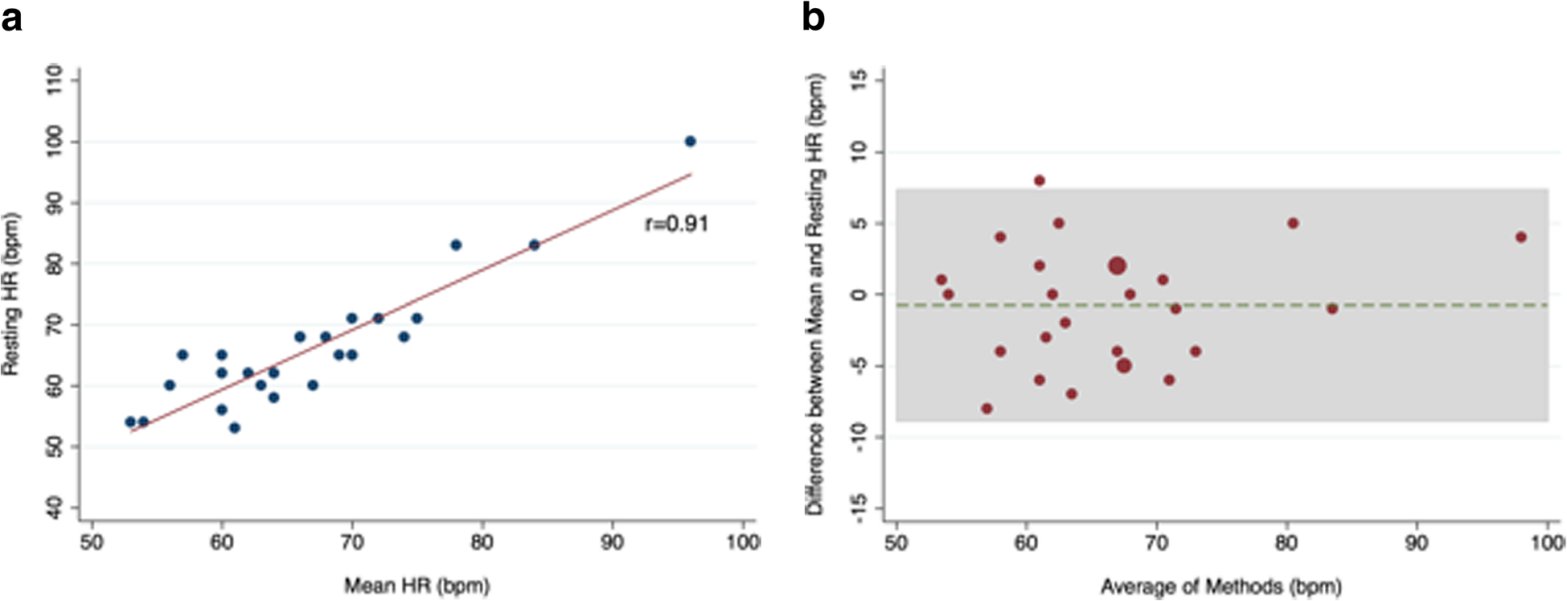
**a** Resting HR/mean HR in the subgroup on an intermediate-to-high dose beta-blocker. **b** Bland-Altman Plot of resting HR/mean HR in the subgroup on an intermediate-to-high dose beta-blocker

We performed a sensitivity analysis for the 39 patients for whom beta-blocker dose was recorded. There was excellent correlation (*r* = 0.91, *p* < .001) between the resting and mean HR amongst patients taking intermediate-to-high dose beta-blockers (Fig. 3a). Bland-Altman analysis demonstrated minimal mean difference (−0.74 bpm) with narrower limits of agreement than for the overall cohort (− 8.9, 7.4 bpm) (Fig. 3b). When the limits of agreement were set to ±5 bpm, 7 (25.9%) of the values fell outside of this range.

### Association between resting heart rate and mean heart rate in subgroup analysis

Since HR and HR variability are influenced by diabetic neuropathy, we performed a sensitivity analysis for the 20 patients with documented diabetes. Amongst this subgroup, the average ECG HR and ICD HR was 69.5 ± 7.6 and 70.2 ± 8.7, respectively. Correlation between resting and ICD HR was moderate (*r* = 0.61, limits of agreement − 15.0, 13.5). We also performed sensitivity analysis for the 16 patients with ventricular tachycardia or nonsustained ventricular tachycardia on interrogation. Correlation was strong with persistently wide limits of agreement (*r* = 0.93, limits of agreement − 8.0, 8.8 bpm). Given that pacing could affect the ICD HR, we performed a sensitivity analysis of the patients paced < 1% in the cardiac chamber from which the mean HR was determined, those paced > 1%, and those with a CRT device in situ (Additional file 3: Table S1). In all three groups the mean difference remained low (0.3, − 0.2, and − 0.7 respectively), the correlation was moderate to strong (0.80, 0.60, and 0.61 respectively), and the limits of agreement were wide (− 13.6, 14.1; − 9.8, 9.4; − 14.2, 12.7 respectively).

### Association between resting heart rate and heart rate interval

To further validate our findings, we sought to determine whether resting HR could predict the HR interval in which a patient spent the most time. Data were categorized by 10-bpm intervals on the histogram and compared with the resting HR. In the overall cohort, 28% (*n* = 15) had a resting HR above the highest % HR interval, 50% (*n* = 27) had a resting HR within this interval, and 22% (*n* = 12) had a resting HR below the highest % interval (Table 2). In the subgroup with HR ≤ 70 bpm (*n* = 36), 22% (*n* = 8) had a resting HR above the highest % HR interval, 47% (*n* = 17) had a HR within this interval, and 31% (*n* = 11) had a resting HR below the highest % HR interval (Table 2).

**Table 2:** Agreement between resting HR and 10-bpm HR interval

	Overall cohort (*n* = 54)		Resting HR ≤ 70 bpm (*n* = 36)	
	*N*	*%*	*N*	*%*
Resting HR higher than highest% HR interval	15	28%	8	22%
Resting HR within highest% HR interval	27	50%	17	47%
Resting HR below highest% HR interval	12	22%	11	31%

*HR* heart rate

## Discussion

In the present, hypothesis generating study, we examined a novel method of mean HR determination from prolonged ICD monitoring. Although our data demonstrated good correlation between resting and mean HR with minimal systematic bias between the 2 methods, the limits of agreement were unacceptably wide. Since there is no gold standard for HR determination we further assessed whether resting HR obtained at a single time point could predict the highest frequency HR interval over the duration of ICD monitoring. Our findings demonstrated that resting HR only reflected the highest frequency HR interval half of the time as determined from the HR histogram. Importantly, resting HR more frequently overestimated the HR interval than underestimated it. From a clinical perspective, this suggests that single time point resting HR is an imperfect surrogate for mean HR when titrating pharmacotherapies.

Our findings of correlation between methods are consistent with other small studies comparing resting and HRs obtained by Holter monitoring. Carlson et al., found partial correlation between resting ECG HR and both mean daytime HR as well as resting HR determined by Holter monitoring in a cohort of otherwise healthy participants [17]. The strength of correlation was weaker than in the present study, which may be explained by the lower activity levels of the patients in our study as well as lower heart rate variability. Moreover, the routine use of beta-blockers in this population would be expected to further contribute as evidenced by the strong correlation between methods in the subgroup on an intermediate-to-high dose beta-blocker. Furthermore, in a telemonitoring study of ICD patients with NYHA class III heart failure, resting HR correlated with mean 24-h HR [18]. More recently Jolly et al., showed a positive correlation between ambulatory Holter mean HR and ECG resting HR in patients with atrial fibrillation [23].

Our study extends the literature by demonstrating that correlation between methods for determining HR does not necessarily imply that the two different approaches yield the same measurements. After confirming correlation, we determined that the limits of agreement between resting single time point HR and mean HR on prolonged monitoring were wide. While there is no consensus on how wide a limit of agreement is acceptable, from a clinical perspective, a 10-bpm difference would be likely to alter clinical decision-making. When the limits of agreement were set to a clinically meaningful interval of ±5 bpm, 37% of patients fell outside of this range. Furthermore, we demonstrated that resting HR was frequently above the mean HR, which is consistent with the findings of Pastor-Perez et al., who identified a subgroup of patients with HF in whom resting HR was above the mean with 7-day ambulatory HR monitoring [24].

The present findings, both using Bland-Altman analysis and comparing resting HR to the highest frequency heart rate interval, raise concern for iatrogenic bradycardia when resting ECG heart rate is used to titrate therapies. This issue was clinically highlighted in both the SHIFT [13] (Systolic Heart failure treatment with the I_f_ inhibitor ivabradine Trial) and SIGNIFY [20] (Study Assessing the Morbidity-Mortality Benefits of the I_f_ Inhibitor Ivabradine in Patients with Coronary Artery Disease) trials where resting ECG was used to measure heart rate. In these trials, the rate of bradycardia in the treatment arm was 10.3% and 18.9% respectively. Thus, uptitrating HR lowering therapies, such as ivabradine, using a resting ECG has the potential for detrimental outcomes particularly given that it has never demonstrated an independent mortality benefit in this population. Clinically this iatrogenic bradycardia may result in failure to augment cardiac output sufficiently to maintain adequate perfusion with ensuing presyncope and falls. Moreover, while there is no consensus on the lower HR limit for a mortality benefit, our group found a non-significant trend towards increased 30-day mortality in a cohort of community-based HF patients with HR < 60 bpm [7]. Furthermore, in the MESA cohort, there was a U-shaped mortality distribution amongst individuals taking HR-modifying therapies with increased mortality at HR < 50 bpm on resting ECG [25]. Finally, in a heart failure population such as ours, who frequently are recipients of an ICD or pacemaker, over titration of medications may result in increased RV pacing which also carries negative prognostic implications [26]. In the context of these concerns, our findings suggest that an alternative method of HR assessment to supplement single time point measurements, should be considered by clinicians. Since many of these patients will have an ICD, either the mean HR or highest frequency HR interval could be used in order to reduce the risk of iatrogenic bradycardia. Long-term studies are then required to assess whether this method will also have prognostic implications.

Our study has several limitations. Firstly, mean HR could not be directly assessed from the ICD, as most commercially available devices do not offer this function. However, we developed a novel method of mean HR determination using the HR histogram which demonstrated strong correlation with resting HR, confirming its validity as a tool to measure HR. Secondly, the presence of tachyarrhythmias such atrial fibrillation or ventricular tachycardia could have influenced the results. With respect to atrial fibrillation, the documented burden was low and only three of these patients had single lead device in which case it would have been difficult to assess the atrial fibrillation burden. The episodes of NSVT/VT were also brief and unlikely to have affected the overall results. Our sensitivity analyses of these subgroups demonstrated results similar to the overall cohort, with moderate to good correlation but wide limits of agreement.

Thirdly, resting HR was taken at a single time point near the end of the clinic visit after the patient had been seated or recumbent and thus, in some instances the patient may have moved or become anxious, thus transiently raising their resting HR. However, this would have been expected to weaken the correlation between the two methods of measurement, which nonetheless remained robust. Fourthly, it is possible that the beta-blocker dose was adjusted during the time between device interrogations. However, it is equally likely that the doses would be increased or decreased. Given that this would result in non-differential misclassification, it would bias the results toward the null and thus is unlikely to affect the finding of the present study.

## Conclusion

In conclusion, while there is good correlation between single time point resting HR and mean HR as determined from the ICD histogram, the limits of agreement are unacceptably wide and resting HR is frequently above the mean. These findings raise concern for the current clinical strategy of using a single time point method of heart rate assessment to titrate negative chronotropic therapies, which may result in iatrogenic bradycardia. Further studies are warranted to address the prognostic implications of these findings and the relative safety of these two methods when titrating chronotropic therapies.

## Additional files

Additional file 1: Figure S1. Mean HR determination from the device histogram. HR, heart rate. (TIFF 159 kb)

Additional file 2: Figure S2. Bland-Altman analysis for the overall cohort with limits of agreement set to ±5 bpm from the bias. (TIFF 294 kb)

Additional file 3: Table S1. Correlation and agreement between single time point and ICD heart rate in the unpaced, paced, and CRT paced subgroups. (DOCX 14 kb)

## Abbreviations

ECG: Electrocardiogram
HR: Heart rate
ICD: Implantable cardioverter defibrillator
LVEF: Left ventricular ejection fraction
