# Supplementary figures and images for: Heart rate in patients with reduced ejection fraction: relationship between single time point measurement and mean heart rate on prolonged implantable cardioverter defibrillator monitoring
Source: BMC Cardiovasc Disord. 2018 Jan 31;18:17. doi: 10.1186/s12872-018-0751-2 (PMC5793357; doi:10.1186/s12872-018-0751-2)

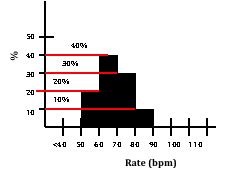

Supplement: Supplementary file 1 — Mean HR determination from the device histogram. HR, heart rate. (TIFF 159 kb) [file 12872_2018_751_MOESM1_ESM.tiff]

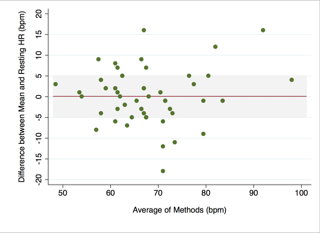

Supplement: Supplementary file 2 — Bland-Altman analysis for the overall cohort with limits of agreement set to ±5 bpm from the bias. (TIFF 294 kb) [file 12872_2018_751_MOESM2_ESM.tiff]
