# Supplementary material for: Heart rate in patients with reduced ejection fraction: relationship between single time point measurement and mean heart rate on prolonged implantable cardioverter defibrillator monitoring
Source: BMC Cardiovasc Disord. 2018 Jan 31;18:17. doi: 10.1186/s12872-018-0751-2 (PMC5793357; doi:10.1186/s12872-018-0751-2)
Supplement: Supplementary file 3 — Correlation and agreement between single time point and ICD heart rate in the unpaced, paced, and CRT paced subgroups. (DOCX 14 kb) [file 12872_2018_751_MOESM3_ESM.docx]

**Additional file 3: Table S1: Correlation and agreement between single time point and ICD heart rate in the unpaced, paced, and CRT paced subgroups**

|  | N | Mean ECG | Mean ICD | R | Mean Difference | Lower Limit | Upper Limit |
| --- | --- | --- | --- | --- | --- | --- | --- |
| Overall | 54 | 67.9±10.1 | 67.8±9.6 | 0.79 | 0.1 | -12.5 | 12.7 |
| Atrial or ventricular pacing<1% | 35 | 70.5±11.1 | 70.3±10.4 | 0.80 | 0.3 | -13.6 | 14.1 |
| Atrial or ventricular pacing >1% | 19 | 63.0±5.3 | 63.2±5.7 | 0.60 | -0.2 | -9.8 | 9.4 |
| CRT | 22 | 69.1±7.7 | 69.8±7.9 | 0.61 | -0.7 | -14.2 | 12.7 |

CRT, cardiac resynchronization therapy; ICD, implantable cardioverter defibrillator; ECG, electrocardiogram
